# Evolutionary Tempo, Supertaxa, and Living Fossils

**DOI:** 10.1093/sysbio/syaf020

**Published:** 2025-05-30

**Authors:** Graham E Budd, Richard P Mann

**Affiliations:** 1 Department of Earth Sciences, Palaeobiology, Uppsala University, SE 752 36 Uppsala, Sweden; 2 Department of Statistics, School of Mathematics, University of Leeds, Leeds LS2 9JT, UK

**Keywords:** living fossils, molecular clocks, patterns of diversification

## Abstract

A relationship between the rate of molecular change and diversification has long been discussed, on both theoretical and empirical grounds. However, the effect on our understanding of evolutionary patterns is yet to be fully explored. Here, we develop a new model, the Covariant Evolutionary Tempo model, with the aim of integrating patterns of diversification and molecular evolution within a framework of a continuously changing “tempo” variable that acts as a master control for molecular, morphological, and diversification rates. Importantly, tempo itself is treated as being variable at a rate proportional to its own value. This model predicts that diversity is dominated by a small number of extremely large clades at any historical epoch including the present; that these large clades are expected to be characterised by explosive early radiations accompanied by elevated rates of molecular evolution; and that extant organisms are likely to have evolved from species with unusually fast evolutionary rates. Under such a model, the amount of molecular change along a particular lineage is essentially independent of its height, which weakens the molecular clock hypothesis. Finally, our model explains the existence of “living fossil” sister groups to large clades that are species poor and exhibit slow rates of morphological and molecular change. Our results demonstrate that the observed historical patterns of evolution can be modelled without invoking special evolutionary mechanisms or innovations that are unique to specific times or taxa, even when they are highly nonuniform.

The relationship between micro- and macroevolution has long been debated (Jablonski, 2000; Erwin, 2000; Rolland et al., 2023). A central question is the extent to which large-scale evolutionary patterns—observed in the fossil record and inferred from phylogenies—are shaped by the processes operating at the population level. Regardless of the outcome of this debate, however, there is often a *methodological* assumption of independence between microevolutionary changes (e.g., shifts in gene frequencies due to selection) and macroevolutionary patterns (e.g., diversification trends within a clade). Contemporary models of evolutionary history conceptualize the overall process as being governed by three independent components: the model of molecular substitution, the rate at which substitutions occur, and the nature of the branching process (Warnock and Wright, 2021). The simplest approach would be to employ a strict molecular clock with a Jukes–Cantor substitution model (Jukes and Cantor, 1969) on a known phylogeny, and assuming a fixed rate of branching—often represented by a homogeneous birth–death process (BDP) (Nee, 2006). Methodological advances, such as the development of relaxed clocks, now allow substitution rates to vary across the tree (see Dos Reis et al. (2016) for a review). Additionally, increasingly sophisticated models of molecular evolution have been introduced (Arenas, 2015). More recently, models have also emerged that incorporate variable diversification rates (see below), allowing for more complex representations of evolutionary trees, although the broad-scale patterns resulting from such models remain relatively unexplored.

Increasing sophistication in modeling ability has naturally also fuelled attempts to understand the causes behind the variation being captured. To take molecular substitution rate variation first: two broad hypotheses exist about its causes. The first encompasses a range from mutational effects to features of the entire organism (such as body size or generation time), and the second is a “speciation rate hypothesis” that links molecular change to speciation (Jobson and Albert, 2002). There are sound empirical and conceptual reasons for thinking that speciation and molecular change may well be intimately related (Hua and Bromham, 2017), and attempts have sometimes been made to consider them jointly (e.g., Sarver et al. (2019); Ritchie et al. (2022b)). Indeed, Eo and DeWoody go so far as to claim that “One of the most basic predictions in evolutionary biology is that the rate of diversification along a particular branch of the tree of life is some function of the rate of genome evolution on that branch.” (Eo and DeWoody (2010), p. 3587). Provocative evidence for a close correlation of the two processes is seen for example in the early history of arthropods (Lee et al., 2013), where early branches of the clade contain just as much molecular change as later branches despite being far shorter in duration (Budd and Mann, 2020b), at least when the tree height is constrained by the fossil record. However, this is just one of several studies that over the last few decades have debated a potential link between both morphological and molecular rates of change and rates of speciation (e.g., Barraclough and Savolainen (2001); Webster et al. (2003); Xiang et al. (2004); Venditti and Pagel (2010); Lanfear et al. (2010); Rabosky et al. (2013); Berv and Field (2018); Bromham (2024)), although it should be noted that not all studies have found clear evidence of this link (e.g., Goldie et al. (2011)). There are at least two factors that might cloud the relationship between diversification and molecular change through time. The first is the so-called “node density” effect, wherein in clades with more terminals, a resulting greater number of internal nodes will recover more molecular change and thus generate a spurious relationship between clade size and amount of molecular change (Hugall and Lee, 2007). The second is that if a relaxed clock methodology is employed to ascertain the time of origin of a clade, then any early burst of molecular (or morphological (Beck and Lee, 2014)) change or indeed diversification is likely to be smoothed out by pushing the age of the root deeper (Bromham, 2003; Beaulieu et al., 2015; Budd and Mann, 2020b; Bromham, 2020; Shafir et al., 2020). If one were simply to accept the result of the molecular clock, then the apparent elevated early rates could theoretically be explained as an artefact caused by “bunching up” the early lineages to artificially squeeze the clade into a too-narrow time interval (c.f., Bromham and Hendy (2000)). However, we have previously marshalled strong reasons for thinking that the fossil record in such instances is often reliable, in which case early bursts of diversification should be taken seriously and not dismissed as dating artifacts (Budd and Mann, 2020a,b, 2024; Holmes and Budd, 2022). As a result, the well-known mismatch between the explicit fossil record and molecular clock origination estimates for many major clades such as animals (Budd and Mann, 2020b), birds (Berv and Field, 2018), placental mammals (Budd and Mann, 2024), and angiosperms (Coiro et al., 2019; Smith and Beaulieu, 2024) itself points to cryptic excess molecular change at the base of trees (Beaulieu et al., 2015; Berv and Field, 2018). Previous critiques of molecular clocks have focused on either inappropriate age priors (e.g., Budd and Mann (2024); Brown and Smith (2018)) or issues with rate heterogeneity (e.g., Bromham and Woolfit (2004); Berv and Field (2018)); below, we will suggest that these are effectively two sides of the same coin. Clearly, if the branching process and rate of molecular change really are correlated, then this would have a significant impact on our understanding of the patterns of evolutionary change through time (see Duchêne et al. (2017) for investigation and discussion of this point).

Causes of variation in diversification rates are likewise much debated (e.g., Moen and Morlon (2014)). It is clear that, similarly to the case of molecular evolution itself, rates of diversification must vary across the tree, as a single homogeneous BDP cannot possibly capture the true patterns of diversification reflected in evolutionary history (c.f., Benton and Emerson (2007)). Notwithstanding this, the homogeneous BDP (Nee, 2006) (in which rates of speciation and extinction are fixed) is still commonly employed in molecular analysis, especially for dating purposes, although its inadequacies are increasingly being recognised (e.g., Khurana et al. (2024)).

Any attempt to investigate a link between rates of genetic/morphological evolution and speciation must reckon with the heterogeneous nature of all of these variables. Historically, rate heterogeneity has largely been addressed in one of two ways: either by assuming rate shifts occur at significant points (e.g., Soltis and Soltis (2016)), or by assuming broad secular variation, for example, with declining rates through time across the entire tree (Strathmann and Slatkin, 1983; Nee et al., 1994b); or some combination of both (e.g., in BAMM (Rabosky et al., 2014)). More recent models have moved away from considering isolated rate shifts to allow rates to vary either in small frequent increments associated with speciations (Maliet et al., 2019; Shafir et al., 2020) or continuously through anagenetic diffusion (Quintero et al., 2024) (for other noncontinuous models, see the review in the supplementary information of Maliet et al. (2019)). The primary goal of these models has been the *inference* of rates through time, based on molecular data from extant taxa (Barido-Sottani and Morlon, 2023) which has now been implemented in BEAST2 (Bouckaert et al., 2019), clearly a substantial step forward from homogeneous models. However, some forward simulation has also revealed that these models can generate clades that match empirical observation; in particular, simulated clades are often imbalanced and “stemmy” (Maliet et al., 2019). This suggests that diversification rate heterogeneity may be one key to understanding the patterns of modern diversity. This is largely because the distribution of modern diversity predicted by homogeneous or epochally time-varying BDPs is geometric (Kendall, 1948; Nee et al., 1994b), and this remains the case even when nonselective mass extinctions are considered (Budd and Mann, 2020a). However, a certain amount of evidence suggests that extant sizes are in fact over-dispersed relative to this expectation (Blum and François, 2006; Stadler et al., 2016). Consider, for example, the crown-group animal phyla, which for the sake of argument, we can assume all emerged around 500 Ma (Budd and Mann, 2024). Estimating total species diversity in the phyla is fraught with difficulty, but even so the species count differs widely. For example, the phyla have an average diversity of c. 50 000 species, but the arthropods have a diversity of well over one million species, thus being over twenty times larger than expected. Under a geometric distribution, this is essentially impossible ($p∼10^{-7}$). This pattern is seen repeated hierarchically: for example, most arthropods are insects, and most insects appear to be hymenopterans (Forbes et al., 2018). Similarly, the angiosperms are much more diverse than any other plant clades (e.g., c. 300 000 vs. 1000 gymnosperms) and birds much more so than crocodiles in the archosaurs (c.10 000 vs. c. 85). In other words, the existence of Stanley’s “supertaxa” (Stanley, 1998) does not seem compatible with a purely geometric distribution of clade sizes as predicted by the homogeneous BDP. In addition, clade sizes show a complex relationship with age that is not easily explained by homogeneous diversification (Magallon and Sanderson, 2001; McPeek and Brown, 2007; Rabosky, 2010), and indeed attempts to estimate absolute diversification rates within a clade suggest several orders of magnitude variation (Magallon and Sanderson, 2001). It thus seems that clade sizes do often appear overdispersed relative to any expected geometric distribution (Khurana et al., 2024).

Taking these empirical findings together, and noting the apparent importance of rate heterogeneity across both microscopic and macroscopic evolutionary scales (Henao-Diaz and Pennell, 2023), it seems that a need exists for a synthesis that unites molecular evolution and species diversification, in which both vary through time. In this paper, then, we develop a model of diversification and molecular change in which all evolutionary rates covary, being controlled by a single variable evolutionary *tempo* that differs both between species, and within a species over time. Although our model does not depend on a particular instantiation of tempo, we nevertheless offer some suggestions about how it might be encoded in a realistic way in the genome below (see schematic for genetic encoding of tempo in Appendix 1). Our analysis of this model will show that it is consistent with the concentration of species into relatively few “supertaxa”’ (Stanley, 1998); that it offers a resolution to conflict between the fossil record and molecular clocks; and that it makes new predictions about the early history of major clades and the fate of the smaller clades that constitute the remaining part of modern diversity. Because of the way we formulate the model, it is amenable to numerical solution that allows us to investigate its general features, as opposed to simulations that would show the outcomes of rates over specific trees.

## Methods and Materials

### Model Outline

As indicated above, heterogeneity in rates of speciation and extinction is key to explaining important empirical features of diversification. We here extend earlier approaches to model such heterogeneity (Rabosky et al., 2014; Maliet et al., 2019; Ritchie et al., 2022a; Quintero et al., 2024) and create a BDP model in which rates of speciation and extinction vary continuously and *covariantly* through anagenetic diffusion. We call this model the Covariant Evolutionary Tempo (CET) model. Under CET, all evolutionary rates are specific to a given taxon at a specific moment in time. Our model is close in formulation to that of Quintero et al. (2024). However, whereas they model this variation in speciation and extinction rates as geometric Brownian motion with an overall drift, and treat speciation and extinction independently, we instead posit that there exist baseline rates of speciation (λ) and extinction (μ) that are linearly modulated by a new variable we label as *tempo*, τ, which controls the relative rates of all evolutionary processes. At any given time, a taxon with tempo τ has a speciation rate τλ and an extinction rate τμ.

This model is fully covariant, in that all rates are linked directly to τ; in effect, the tempo represents a local speeding-up or slowing-down of evolutionary time, such that all processes happen faster or slower. In particular, we posit that tempo *itself* varies through time, and because we posit that tempo is in some way genetically encoded, this implies that the evolution of τ itself proceeds at a rate proportional to τ, since the effect on molecular rates of mutation will obtain upon whichever part of the genome is responsible for this encoding. Specifically, we model the log-tempo (x= log ⁡ τ) as evolving according to a modified Ornstein–Uhlenbeck (OU) process that incorporates the effect of the tempo itself on all rates


$$\begin{array}{ccc} dx=-\theta e^{x}xdt+\sqrt{2\theta s^{2}e^{x}}dW, & & \end{array}$$ (1)

where dW represents an incremental change from a Wiener process (popularly known as Brownian motion). We impose this model for the evolution of the log-tempo x since the tempo itself is constrained to be positive. The parameters of this stochastic differential equation are the mean reversion rate θ and the stationary variance of the process, $s^{2}$. The $e^{x}$ terms in this equation come from the self-interaction of the tempo, which as well as multiplying the rate of all other processes also determines the rate at which it evolves itself, such that the effective increment of time is $\tau dt=e^{x}dt$. Our use of an OU process is motivated by two considerations. First, as we shall show, a Wiener process without a restoring force would lead to a runaway effect, where tempos increase without limit. Secondly, in Appendix 1, we describe a plausible schematic for how tempo is inherited that produces an inherent reversion to a mean value via entropic forces.

As we show in Appendix 1, this results in a drift-diffusion partial differential equation for the generating function of the resulting BDP


$$\begin{array}{ccc} \frac{\partial G_{x}}{\partial t}=e^{x}\left((\lambda G_{x}-\mu )(G_{x}-1)-\theta x\frac{\partial G_{x}}{\partial x}+\theta s^{2}\frac{\partial^{2}G_{x}}{\partial x^{2}}\right), & & \end{array}$$ (2)

where $G_{x}(t,z)=\sum_{n=0}^{\infty }P_{n}(t,x)z^{n}$, with $P_{n}(t,x)$ being the probability of generating n species over time t in a process starting with log-tempo x. Solving this equation for an initial condition $G_{x}(t=0,z)=z$ provides the value of the generating function $G_{x}(t,z)$.

Equation 2 does not appear to permit solution in closed form, except for the long-term extinction probability $G_{x}(t,z=0)$ for t→∞, which is $\frac{\mu }{\lambda }$ for all x, and is therefore tempo invariant. More generally, equation 2 can be straightforwardly solved numerically. The values of $P_{n}(t,x)$ can be retrieved from this generating function by Fourier inversion (see Appendix 1).

We can derive further equations specifying the evolution of the mean number of species generated by the process over time, the expected number of lineages (species that will have modern descendants), and the distribution of tempos over time. Derivation of these equations is described in Appendix 1. The most important of these equations specifies the evolution of the mean number of species through time. Given a generating function $G_{x}$, the mean of the distribution $N_{x}(t)\equiv E(n|t,x)$ is given by


$$\begin{array}{ccc} N_{x}(t)=\frac{\partial G_{x}(t,z)}{\partial z}{|}_{z=1}. & & \end{array}$$ (3)

Using this relation, equation 2 can be transformed into a simpler, linear form to represent the dynamics of the mean


$$\begin{array}{ccc} \frac{\partial N_{x}}{\partial t}=e^{x}\left(rN_{x}-\theta x\frac{\partial N_{x}}{\partial x}+s^{2}\theta \frac{\partial^{2}N_{x}}{\partial x^{2}}\right), & & \end{array}$$ (4)

where r=λ−μ is the baseline net diversification rate. This equation reveals the key dynamics of the process: the expected number of species with log-tempo x locally increases exponentially at the rate r modulated by $\tau =e^{x}$. At the same time, a drift-diffusion process modifies the tempo of each species, such that species tend to move toward a log-tempo of 0 (i.e., τ=1).

### Justification for a Covariant Theory

Why should all evolutionary rates be covariant? As we have discussed above, previous birth–death models have allowed for independent variation in speciation and extinction (while in practice sometimes holding one of these constant), while the rates of molecular evolution have been assumed (generally implicitly) to be completely independent of diversification rates. In one sense, our choice is pragmatic: we seek to explore the consequences of linking changing rates of molecular evolution to diversification rates, and the most parsimonious way to do this is to impose a perfect correlation between the two. Allowing for speciation and extinction rates to vary independently (or with some nonunitary correlation) would greatly complicate the mathematical formulation of the birth–death model and its analysis, and cloud its implications. Empirically, we are also strongly motivated by the apparently close (inverse) correlation between rates of molecular evolution and branch durations in for example Lee et al. (2013) and other studies, as noted in the introduction. Finally, our choice is also theoretically informed. It is clear that as speciation and extinction vary, they must remain close to one another over time; a sustained period of much higher speciation will quickly produce an unrealistically large number of species, while a period of greater extinction than speciation will almost certainly drive the clade to extinction. Indeed, the linkage between the two has been formulated by Marshall as the third of his five “paleobiological laws” (see Marshall (2017) for discussion and justification of this point). Moreover, we expect that rates of speciation and extinction may largely be driven by the same causal factors, for example, generation times and population size (for a classical discussion of the various links between speciation and extinction rates, see Stanley (1990), and more recently Greenberg and Mooers (2017)). Therefore, while we anticipate significant deviations from covariance between these processes at sufficiently short time scales, we expect it to be a realistic first-order approximation when considering rates on the scale of millions of years. We also note that although most discussions of molecular evolution have considered a link with speciation, we consider that in practice, this implies a link with extinction too, for the reasons given above.

As far as our model is concerned, we note that many of the factors operating on speciation rates are also likely to affect molecular rates of change. For example, Bromham has stressed the need to consider the genome itself as a life-history trait ((Bromham, 2003, 2009, 2020), and thus open to the same influences [population size, generation time, etc]) as other traits. Thus, under such a view of evolution, small body size or small populations might both influence speciation rate (Martin, 2017; Cooney and Thomas, 2021) and molecular evolution rates (Bromham, 2020) together, thus uniting the two broad ways of considering the causes of molecular change (Jobson and Albert, 2002). Naturally, such a linkage between the two might itself vary, but in order to investigate its general effects, and certainly to greatly simplify the analysis, we have chosen a model with complete linkage.

Few studies have shown a convincing direct link between molecular substitution rates and phenotypic change (Bromham and Woolfit, 2004). Nevertheless, the two may be indirectly linked by other factors such as speciation rate, as both phenotypic and molecular are plausibly linked to speciation (for discussion of this point with some examples such as placental mammals and lungfish, see Budd and Mann (2018)). As we suggest below, some empirical evidence points to this being true, at least in some clades.

## Results

We analyzed our model by solving the probabilistic equations given above to obtain distributions at different time epochs, rather than by direct simulation of the tree evolution. Notably, our analysis does not provide a probability distribution over specific trees, but over coarser-grained variables such as diversity. It is not our goal to quantitatively fit our model to the modern diversity or evolutionary history of any specific clade, but rather to reveal the qualitative features the model predicts. Throughout we use a core set of parameters λ=0.51 per species per myr, μ=0.5 per species per myr, θ=0.01/myr, and s=1. These parameters are chosen to reflect reasonable expectations about the real evolutionary process: a baseline extinction rate of μ=0.5 per species per myr comports with that chosen in previous analyses (e.g., Budd and Mann (2018)) and, combined with a speciation rate of λ=0.51 per species per myr is consistent with a typical species existing for c. 1 myr, in broad agreement with the fossil record (see. e.g., Budd and Mann (2018)). The speciation rate is chosen to be of similar magnitude to the extinction rate, such that extinction plays a significant role in the evolutionary dynamics (Marshall, 2017) but is otherwise arbitrary. We choose a mean-reversion parameter θ=0.01/myr to be equal to the net diversification rate as we will later show that if r=θ, then the mean log-tempo converges to 0 (see Appendix 1, equation A28). Although this choice is mathematically convenient, we do not expect that it represents any necessary feature of the evolutionary process nor do the general features of our results depend on it. Finally, the diffusion parameter s=1 is chosen to be large enough to produce significant effects of the diffusive dynamics, and otherwise is simply a mathematically convenient choice.

### Distribution of clade sizes

**Figure 1:**
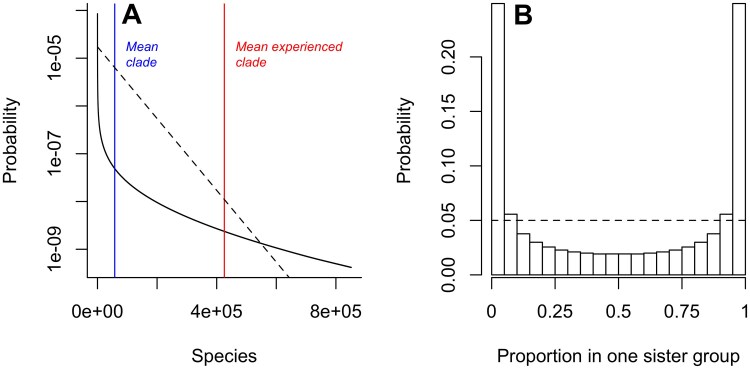
A) Distribution of the number of species generated in clades that survive 500 myr, with parameters λ=0.51 per species per myr, μ=0.5 per species per myr, θ=0.01/myr ,s=1, and an initial log-tempo x=0. Note the log scale on the *y*-axis. The distribution is long-tailed and is characterized by a high probability of few species (P(n<1000)≃1∕3) and a long tail allowing some very large clades to be generated (P(n>50000)≃1∕4). The blue and red lines indicate the mean clade size (c. 60 000) and the mean experienced clade size of a randomly chosen taxon (c. 400 000) respectively, indicating that most taxa are found in very large clades. The dashed line shows the geometric distribution with the same mean expected under a standard BDP. B) The probability distribution for the proportion of diversity contained within one randomly chosen sister group of a crown group, indicating that clades are typically highly imbalanced, with one sister group being much larger than the other. The dashed line shows the uniform distribution expected under a standard BDP.

We solved equation 2 for times 0≤t≤500 myr and starting log-tempos −10<x<10 and performed a Fourier inversion (see Appendix 1) to retrieve the implied probability distribution Pn(t=500 myr,x). The distribution of clade sizes for a clade that starts with log-tempo x=0, excluding clades of size zero, is shown in Figure 1A. The clade sizes follow a distribution that differs strongly from the geometric distribution expected under a typical BDP (indicated by the dashed line, assuming the same mean clade size). This distribution is characterised by most clades being small, but with a few extremely large clades. This means that clades that are many times greater than average (either mean or median) are much more probable than under a standard BDP. A corollary of this is that clade size a typical species “experiences” (i.e., the expected clade size of a randomly selected species) is c. 8 times greater than the mean clade size. For clarity, we here define the experienced and mean clade sizes as the sizes of clades containing living organisms that have the same time of origin (e.g., the sizes of parent clades that are all 500 myr old).

In Figure 1A, we indicate both the mean clade size and the mean experienced clade size for illustration. This result should be compared to the equivalent result from a standard BDP where the mean experienced clade is only two times greater than the mean (Budd and Mann, 2018). This implies that the large majority of species we might encounter and/or study are contained in extremely large clades. Since clades are hierarchically structured this also implies that the diversity of any clade is likely to be dominated by its largest sub-clade. To illustrate this, we consider the two sister-groups of a clade originating 500 Ma and calculate the expected proportion of the total diversity that is contained in one sister-group chosen at random. As shown in Figure 1B, the probability that a given proportion of total diversity is contained in a given sister-group is peaked strongly close to zero and one, indicating that one sister-group or the other typically contains the large majority of species in the clade as a whole. For example, there is a c. 50% chance that the larger sister group is at least 20 times larger than the other. This can be compared with the equivalent result under a standard BDP, in which the proportion of diversity contained in one sister-group is uniformly distributed between zero and one indicated by the dashed line), and thus, the probability of such an imbalance is only 10%. This implies that diversity among clades of the same age tends to follow the Single Big Jump principle (Vezzani et al., 2019), whereby sums of heavy-tailed random variables are dominated by their largest component.

### Diversification Through Time

**Figure 2:**
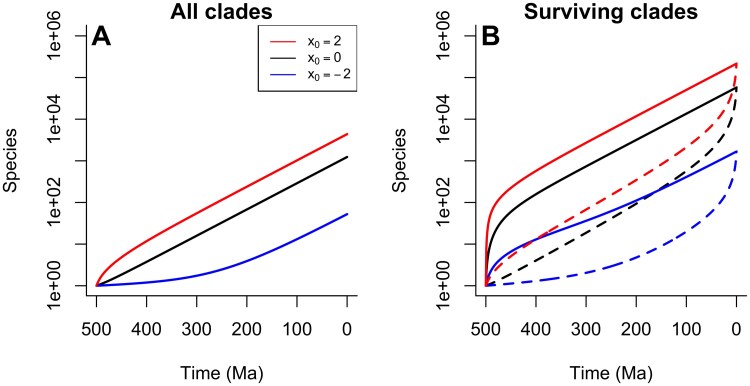
Diversification through time as a function of starting tempo. A) The expected number of species through time for $x_{0}=-2$ a clade starting 500 Ma with different initial log-tempos: $x_{0}=-2$ (blue line); $x_{0}=0$ (black line); $x_{0}=2$ (red line). These expectations include clades that are extinct. Clades with a higher starting tempo initially diversify more quickly (on average); eventually diversification stabilizes to a fixed rate independent of the starting tempo. B) Expected diversification profiles for clades that survive to the present day. Solid lines indicate the expected number of species through time; dashed lines indicate the expected number of lineages—species with surviving descendants. Surviving clades of all starting tempos experience the Push of the Past, mirrored by the Pull of the Present in the lineages (Nee et al., 1994a). This effect is especially pronounced in the clades starting with the highest tempo.

The above analysis reveals the expected pattern of diversity in clades of a fixed age (500 myr) which all start from a common ancestor with a typical tempo (x=0). How does this pattern change through time, and between clades with different initial tempos? To explore these questions, we focused on how the expected clade size varies through time for different initial values of x. We numerically solved equation 4 to obtain the expected clade size as a function of time values 0<t<500 myr, and for different initial values of $x_{0}\in \{-2,0,2\}$. In Figure 2A, we show how the mean clade size varies through time for different initial tempos including clades that have gone extinct before the time in question. In Figure 2B, we show the variation in the mean number of species through time conditioned on knowing that the clade survives to the present day (solid lines), and also the expected number of *lineages* (dashed lines) through time—these are species that have at least one descendant in the present day, and form the “reconstructed process” that can (in principle) be inferred from modern molecular data. Clades that survive to the present experience the “Push of the Past” (Budd and Mann, 2018), an initial period of increased diversification when the clade is small.

These results show that the initial tempo has a substantial impact on how the clade diversifies and its eventual expected size. As we would intuitively expect, clades with high tempos initially diversify more quickly, and conversely, those with low tempos diversify slowly. However, after some period of time, the rate of diversification becomes stable; initially, high-tempo clades slow down and initially low-tempo clades speed up, such that all clades eventually diversify at the same fixed rate, as seen in emergence of parallel lines of growth from all 3 initial conditions.

The tempo of the root node of a clade therefore has transient effects that eventually decay as new species emerge whose own tempos diffuse away from the initial state. The duration of these transient effects is longer in clades that start with low tempos, since all processes including those that control the diffusion of tempos over time run slower. Although the effect of initial tempo is transient, it leaves an important signature in the eventual size of clades over the long term: because initially high tempo clades diversify more quickly in their early history, they reach a larger size before reverting to a constant diversification rate, meaning that they have a much greater expected diversity in the present. This intuitively suggests that the largest clades of a given age in the present are likely to be those that originated from a high-tempo common ancestor.

### Distribution of Tempos Over Time

As a clade diversifies, the various taxa will develop different tempos as they diverge independently from the initial starting tempo, leading to a time-dependent distribution of log-tempos p(t,x). In Appendix 1, we show that the evolution of this distribution obeys a replicator-mutation equation


$$\begin{array}{ccc} \frac{\partial p}{\partial t}=rp\left(e^{x}-\langle e^{x}\rangle \right)+\theta \frac{\partial xe^{x}p}{\partial x}+s^{2}\theta \frac{\partial^{2}e^{x}p}{\partial x^{2}}, & & \end{array}$$ (5)

where the term $\langle e^{x}\rangle =\int_{-\infty }^{\infty }e^{x}p(t,x)dx$ indicates the average value of $e^{x}$ at a given time.

**Figure 3:**
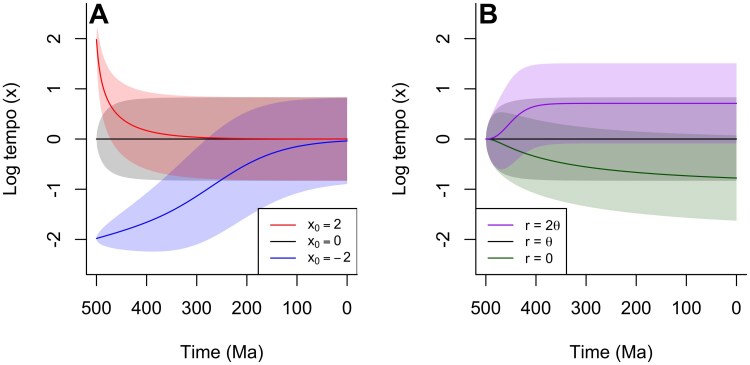
A) The evolution of the distribution of log-tempos through time for clades starting from different initial log-tempos: $x_{0}=-2$ (blue line); $x_{0}=0$ (black line); $x_{0}=2$ (red line). Lines indicate the expected log-tempo of a randomly chosen species, and shaded areas represent the standard deviation. Regardless of starting tempo, clades converge to the same equilibrium distribution of log-tempos. This convergence is fast in clades that start with high tempos. B) Evolution of the log-tempo distribution for clades with different values of the diversification parameter r, with a fixed value of θ=0.01/myr. Starting from the same tempo ($x_{0}=0$), clades reach different equilibrium log-tempo distributions depending on the value of r; higher values of r produce higher average tempos.

We numerically integrated this equation through times 0<t<500 myr for 3 initial starting log-tempos: $x_{0}\in \{-2,0,2\}$ specified by initial conditions of the form $p(t=0,x)=\delta (x-x_{0})$, where δ(⋅) is the Dirac delta function (Shutovskyi, 2023). The resulting evolution of the log-tempo probability distributions is shown in Figure 3. These results show that regardless of the starting tempo of the process, our model converges over time to the same stable distribution of log-tempos that is approximately normally distributed. Using the core set of model parameters described earlier gives a mean log-tempo of zero. When the process is initiated with a high tempo (x=2), the convergence to this stable distribution is very rapid (red line). This is because the initially high tempo forces all processes to run fast, so time is effectively compressed. Conversely when the process is initiated with a slow tempo x=−2, the convergence is much slower, potentially taking hundreds of millions of years. In practical terms, this predicts the existence of long-lived substructures of the evolutionary tree in which evolution is effectively “running slow.” If other evolutionary processes such as molecular and morphological change are also covariant to the tempo, this would imply the existence of lineages with low diversity and minimal morphological or molecular change over very long periods of time. Since such small clades are common (Figure 1A), we expect that these “living fossils” will be ubiquitous, and in particular, that they will often be the sister group to the few large clades that dominate total diversity.

Varying the parameters of our model produces changes in the stable distribution of tempos. In particular, the mean of this distribution increases with larger r and decreases with larger θ (Figure 3B); in the limiting case where r=0, the mean log-tempo can be shown to converge to −1 in closed form (see Appendix 1, equation A26). The dynamics of diversification tend to elevate the mean tempo, since higher tempo lineages produce more descendants on average per unit time, which inherit the same high tempo from their parent nodes. An interesting corollary to this point is that without any sort of mean reversion process, tempos (and thus diversification rates) would simply tend to rapidly increase without limit. As this is not observed empirically, the suggestion must be that something tends to draw log-tempos towards a characteristic mean value (c.f., Aris-Brosou and Yang (2003); Lepage et al. (2006); Maliet et al. (2019)). In Appendix 1, we show that such a mean reversion can arise without implying any necessary ecological mechanism: if tempo is encoded genetically, then intermediate tempos are consistent with a greater number of possible genetic configurations, such that random mutations tend to cause a drift toward these values.

### Patterns of Historical Tempo

So far, we have considered what happens to various features of the evolutionary process as it is run forward from a particular initial condition. However, evolutionary analysis can be considered to be retrospective as well: one attempts to identify and explain patterns of evolution looking back in time from a vantage point in the present. As discussed by Budd and Mann (2018), this perspective necessarily distorts the patterns we are likely to observe, especially if one also chooses to analyze clades that have unusual modern-day properties. Such choices are commonplace: the most studied clades are often unusually diverse relative to clades of similar age; since most species are contained in these large clades, they are often taken to be particularly representative of a particular epoch, despite in fact being a highly unrepresentative sample of clades in general.

To investigate the role that contingencies of clade selection have on the observed patterns of evolution, we considered 2 questions. First, if one randomly selects a species in the present and traces its lineage back in time, what expectations should we have about the evolutionary tempo of those ancestors? Second, what expectations should we have about the average tempo of earlier members of that clade overall? These are different questions since most historical taxa, even those with modern descendants (the lineages) will contribute little to modern diversity, owing to the Single Big Jump Principle (Vezzani et al., 2019) identified earlier (c.f., Figure 1B). That is, the ancestors of most modern taxa constitute a very small subset of historical diversity.

First, we consider how likely it is that a species alive today at time T originated from an ancestor at time t with log-tempo x. Since we assumed that the clade originates with an ancestor drawn from the equilibrium distribution p(x), the prior probability that a species alive at time t has log-tempo x remains p(x) by definition of the equilibrium. We can determine a posterior estimate for the ancestor’s log-tempo by application of Bayes’ rule


$$\begin{array}{ccc} \begin{array}{ccc} p(x & |\mathrm{ancestor of random modern taxon}) & \\ & =\frac{p(\mathrm{ancestor of random modern taxon}|x)p(x)}{p(\mathrm{ancestor of random modern taxon})}. \end{array} & & \end{array}$$ (6)

The likelihood term in this equation, p(ancestor of  random modern taxon∣x) is proportional to the expected number of modern species that an ancestor at time t will generate, $N_{x}(T-)$. This means we can rewrite the above as


$$\begin{array}{llll} & p(x|\mathrm{ancestor of random modern taxon}) & & \\ & =\frac{p(x)N_{x}(T-t)}{\int_{-\infty }^{\infty }p(x^{\prime })N_{x^{\prime }}(T-t)dx^{\prime }}. & \end{array}$$ (7)

The equation above estimates the log-tempo of a direct ancestor of a modern taxon. We can also ask what the tempo of a randomly chosen member of the clade in the past is. To estimate this, we consider the probability of generating $n_{T}$ species at time T from any starting log-tempo (based on solution of the generating function $G_{x}$) and the probability that a randomly chosen species at time t has log-tempo $x_{t}$ if the process starts at $x_{0}$, $p(x_{t}|x_{0})$. From these probabilities, we can infer the probability of a historical log-tempo $x_{t}$ conditioned on the current diversity $n_{T}$, using Bayes’ rule and marginalizing over the unknown starting log-tempo $x_{0}$


$$\begin{array}{lll} \begin{array}{lll} p(x_{t}|n_{T}) & =\int_{-\infty }^{\infty }p(x_{t}|x_{0},n_{T})p(x_{0}|n_{T})dx_{0} & \\ & =\frac{\int_{-\infty }^{\infty }p(x_{t}|x_{0})P(n_{T}|x_{0},x_{t})p(x_{0})dx_{0}}{P(n_{T})}\\ & ≃\frac{\int_{-\infty }^{\infty }p(x_{t}|x_{0})P(n_{T}|x_{0})p(x_{0})dx_{0}}{P(n_{T})}, \end{array} & & \end{array}$$ (8)

where the final approximation assumes that $P(n_{T}|x_{0},x_{t})≃P(n_{T}|x_{0})$. In general, this approximation will be reasonable, because of the earlier result that modern diversity arises from a small subset of historical taxa. If the historical number of species at time t is high, a randomly chosen taxon is unlikely to contribute significantly to modern diversity and we can therefore treat $n_{T}$ as being independent of this species and its tempo. Because of the Push of the Past (Budd and Mann, 2018), surviving clades will rapidly reach this state, and in the special case where t=0 (i.e., the origin of the clade), the approximation holds exactly.

**Figure 4:**
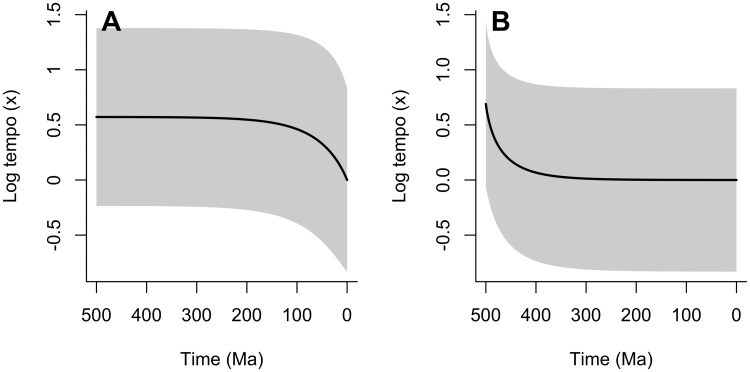
Expected patterns of historical tempo evolution. Lines indicate the expected log-tempo and shaded areas represent the standard deviation. A) The expected historical log-tempo of ancestors of a randomly chosen modern taxon, following its lineage back to the origin of the clade. Throughout this lineage, expected log-tempos are elevated relative to the present day, declining rapidly shortly before the present. B) Expected historical log-tempo of species in the clade as a whole. This is shown for a clade of the mean experienced clade size (the typical clade size of a randomly chosen modern species). Expected tempos are highest at the origin of the clade and decline through time as the clade diversifies. In both panels, the distribution of tempos at time = 0 Ma represents the equilibrium distribution derived as the stable solution to equation A24.

Figure 4 illustrates our expectations about the historical patterns of tempo. Figure 4A shows the distribution of log-tempos for ancestors of a randomly chosen modern taxon, conditioned on our standard set of parameters (λ=0.51 per species per myr, μ=0.5 per species per myr, θ=0.01/myr, s=1). In the present, these are centered around x=0, which is the stable overall distribution of log-tempos shown in Figure 3A. As we look backward in time the expected log-tempo of the ancestor rises sharply, before plateauing at x≃0.6 at c. 100 Ma. While the uncertainty represented by the standard deviation in gray permits a wide variety of ancestral tempos, beyond 100 Ma, these ancestors will have elevated tempos with very high probability. Conversely, the tempo of the clade as a whole tends to peak at its origin, as shown in Figure 4B. This illustrates the overall expected log-tempo of historical species within a clade inhabited by a typical modern taxon (i.e., one with a diversity equal to the mean experienced clade size). That is, the clades that contain most modern taxa are defined by a high early rate of evolution, which then undergoes a consistent secular decline to the present, while the direct ancestors of most modern taxa have uniformly elevated rates of evolution across the history of the clade until close to the present. A consequence of this result is that most modern taxa share relatively recent common ancestors (c. 100–150 Ma), as they overwhelmingly tend to originate via a small subset of lineages that maintain high tempos until this point. This is despite the most recent common ancestor of *all* species being close to the origin of the clade (in other words; the crown group is expected to emerge soon after the total group—for analysis see, e.g., Budd and Mann (2018)).

### Effect of Tempo Variation on Branch Lengths and Duration

We have now considered the effect of tempo variation on the dynamics of the BDP, and by extension on diversification. We motivated our approach by noting that rates of molecular evolution are commonly assumed to vary in modern relaxed molecular clock analyses, and now, we turn our attention to the interaction of molecular evolution and diversification. Specifically, we consider the expected duration (in real time, equivalent to branch height) and amount of molecular change along branches (= branch length) with differing initial log-tempo values. In our model, tempo can vary within a branch, so the duration of branches is not necessarily exponentially distributed, in contrast to standard BDP models. Instead, the probability that a branch terminates (either by speciation or extinction) in a small interval of time Δt depends on its current log-tempo and is given by $e^{x}(\lambda +\mu )\Delta t$.

**Figure 5:**
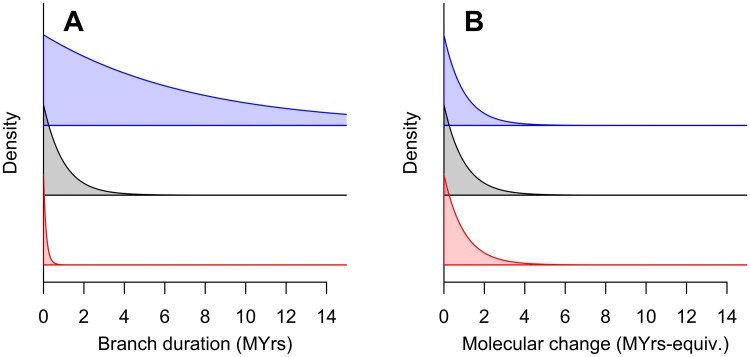
The distribution of branch durations A) and amounts of molecular change along branches B) for branches starting with different log-tempos: $x_{0}=-2$ (blue); $x_{0}=0$ (black); $x_{0}=2$ (red), assuming that molecular evolution is covariant with tempo. Branches that start with lower tempos are much longer on average in real time than those with high tempos. However, the expected amount of molecular change is independent of the starting tempo. 1 myrs-equivalent is the expected molecular change in 1 myrs at a fixed tempo of τ=1.

As shown in Appendix 1, this implies that the probability density $f_{x}(t)$ that a branch originating with log-tempo x terminates at time t obeys a partial differential equation of the form


$$\begin{array}{ccc} \frac{\partial f_{x}}{\partial t}=e^{x}\left(-(\lambda +\mu )f_{x}-\theta x\frac{\partial f_{x}}{\partial x}+\theta s^{2}\frac{\partial^{2}f_{x}}{\partial x^{2}}\right), & & \end{array}$$ (9)

with initial condition $f_{x}(t=0)=-e^{x}(\lambda +\mu )$. Figure 5A shows the solution to this equation for 3 different values of x∈{−2,0,2}, illustrating the intuitive result that branches with lower initial tempos tend to have a greater duration—that is they exist for a longer time before either speciating or going extinct.

How does this effect of the initial tempo translate into the amount of molecular change that occurs within a branch? This is an important question, because the relationship between branch duration and molecular change is fundamental to the practice of molecular dating and potentially more broadly to the inference of phylogenetic relationships based on the molecular genetic data from modern taxa because of the problems caused by long branch attraction (Shafir et al., 2020; Kapli et al., 2021).

If we assume that rates of molecular change covary with tempo alongside all other rates, then the amount of molecular change Δw that occurs in some small unit of time Δt is given by


$$\begin{array}{ccc} \Delta w=e^{x}\Delta t\Rightarrow \frac{dw}{dt}=e^{x}. & & \end{array}$$ (10)

Applying a change of variables to express Equation 9 in terms of the molecular change w gives an equation obeyed by the probability density of molecular change $f_{x}(w)$ in a branch that starts with log-tempo x


$$\begin{array}{ccc} \frac{\partial f_{x}}{\partial w}=\left(-(\lambda +\mu )f_{x}-\theta x\frac{\partial f_{x}}{\partial x}+\theta s^{2}\frac{\partial^{2}f_{x}}{\partial x^{2}}\right), & & \end{array}$$ (11)

with initial condition $f_{x}(w=0)=\lambda +\mu$. Noticing that the partial derivatives in this equation will remain zero for all values of w, this simplifies to a standard exponential distribution


$$\begin{array}{ccc} f_{x}(w)=(\lambda +\mu )e^{-(\lambda +\mu )w}. & & \end{array}$$ (12)

That is, the amount of molecular change contained in a branch is *independent* of the value of the tempo. This is illustrated in Figure 5B.

The key result then is that branches that start at higher tempos are typically shorter, but contain just as much molecular change, as longer branches that originate from lower tempos. This implies that a clade that starts with a high tempo is likely to be characterized in its early stages by short-duration branches that nonetheless contain just as much molecular change as later branches that are longer in duration. Since we have shown above that early high tempos are expected especially in clades that are particularly large, we can expect this pattern to be commonly observed. As a corollary, if we further assume that morphological change also covaries with tempo (c.f., Omland (1997); Lee et al. (2013)), then the same pattern of rapid change along short early branches would be observed morphologically by an analogous argument.

## Discussion

We have described the CET model of macroevolution that allows the rates of speciation, extinction, and molecular/morphological evolution to coevolve through a variable evolutionary tempo parameter. This model provides a resolution to several outstanding difficulties in reconciling classical birth death models with empirical data. Allowing for tempo variation produces much greater variation in clade sizes over a given time horizon than under homogeneous models, consistent with the fact that modern diversity is dominated by a relatively small number of very large clades across different taxonomic levels. An underappreciated consequence of this distribution is that if we wish to understand how modern patterns of diversity arose, it is important to study the characteristic behavior of such large clades, which, as we have shown here, differs markedly from that of clades as a whole. In other words, large and arguably charismatic clades such as arthropods, birds, and angiosperms that are the subject of understandable interest have quite different patterns of evolution than what an “average” clade might be inferred to have.

Our anaylsis predicts that these clades containing the bulk of modern diversity are likely to result from very high early evolutionary tempos, leading to short early branches (measured in real time). Because we conjecture that evolutionary tempo affects all rates in a covariant fashion, these short early branches are nonetheless expected to contain as much molecular and morphological change as later, longer branches, because the rates of molecular and morphological change are elevated in direct proportion to speciation and extinction.

This offers an explanation for the observation of, for example, such elevated rates coupled in short early branches found in molecular studies that take the fossil record as a reliable guide to the age of the clade (e.g., Lee et al. (2013)). In that example, early rates seem to be ∼10 times higher than later ones, which would give a log ⁡ (x) value of 2.3, in a clade that is at least 20 times larger than average. Our initial value of log ⁡ (x) of c. 0.7 for a clade c. 8 times larger than average in Figure 4B seems to be broadly compatible with this. We note that such studies tend to indicate a much older origin of the clade when the firm calibration based on the fossil record is removed; this emerges because of the use of a model that assumes a homogeneous BDP as the underlying description of diversification (Budd and Mann, 2024), and because of a questionable assumption that the processes of diversification and molecular evolution are independent. Modern molecular clock analyses typically employ a “relaxed-clock” methodology that permits substantial changes in the rate of molecular evolution across time and between lineages, but these rates are decoupled from the rates of speciation, extinction, and lineage creation (e.g., Aris-Brosou and Yang (2003)). Such a rigorous decoupling between evolutionary processes seems intuitively unrealistic, and indeed, elevated rates of molecular evolution have been posited as a *cause* of radiations (Lancaster, 2010), while in the fossil record, morphological change is (necessarily) the key signature of diversification. As such, we argue that recognizing the likely covariance between these rates is key to understanding apparent discrepancies between molecular signatures of diversification and the fossil record. Nevertheless, our model does not rely on any particular causal relationship between molecular change and diversification, and indeed, these variables may be linked by underlying factors such as body size (Berv and Field, 2018).

A covariant process that extends to rates of molecular evolution will produce similar amounts of molecular change on all branches of the tree, regardless of their duration in time. This suggests that from a molecular standpoint, there will be little or no difference between an older tree whose branch rates exhibit no secular trend, and a younger tree that experiences rapid early evolution and diversification followed by a slowdown (or indeed an even older tree that experienced very slow early evolution, although these will typically represent only a small proportion of modern diversity). As such, molecular data from modern taxa are unlikely to be able to discern which of these scenarios led to the molecular and species diversity we observe today. Precise and reliable fossil calibrations, in combination with molecular data, can potentially reveal the typical distribution of rates within the time scope of those calibrations. However, extrapolation of younger rates into deeper time is problematic, as we have shown that these are likely to be higher in the past, beyond the deepest precise calibrations (c.f., Budd and Mann (2020b)). This imposes a currently insurmountable barrier to the use of the molecular clock for providing reliable clade age estimates, unless one can argue that rates of speciation and extinction are substantially decoupled from the process of molecular change. As noted earlier, making such an argument would preclude many putative explanations for observed rapid radiations, as well as being counter-intuitive. Although we have analyzed a model in which there is a perfect correlation between all evolutionary rates, in practice, we expect that any significant coupling will severely hamper the use of current clock methodologies. We suggest therefore that the use of molecular clocks for making extrapolative deep-time age estimates is fundamentally unreliable (interpolations within a tree, between nodes of known age are likely to be more constrained, but here, we expect that molecular data will add little to dates derived directly from fossils (e.g., Brown and Smith (2018)).

As well as revealing the broad outlines of the dynamics of a varying tempo model of evolution, our analysis of this model also provides several empirical predictions:

1. Analysis of clades which are known to originate at similar times will show that the large majority of modern diversity is contained in a small subset of these clades. Most concretely, we anticipate that in pairs of sister groups, one group is likely to greatly dominate the diversity of the total (c.f., Aldous (2001)).
2. The smaller sister group in a clade will be that which also experiences lower aggregate molecular and morphological change over its history. As such, the species in this group will tend to retain more plesiomorphic features relative to those in the larger sister group. Potential examples of such a phenomenon include the onychophorans relative to arthropods, cyclostomes relative to gnathostomes (Yu et al., 2024), or priapulids relative to other ecdysozoans (e.g., Webster et al. (2006)). This prediction gives some succour to the popular notion of “living fossil” that are slow-evolving, have few species, and which to some extent resemble ancestral taxa (c.f., Crisp and Cook (2005) for the traditional view that “basal,” species-poor groups should not be regarded as ancestral or “primitive”; and Jenner (2022) for a more general discussion of the issue).
3. The direct ancestors of most modern species will show elevated rates of evolution (diversification, molecular, and morphological) throughout their history. Those lineages that gave rise to a majority of modern species will therefore show consistent rates of molecular evolution until close to the present, when they fall. However, if one analyses all historical taxa in a large clade (which is where most modern taxa reside), we expect to see very high rates of molecular change concentrated at the origin of the clade, declining consistently to the present. Nevertheless, both of these expected patterns take place within a wider context in which rates of evolution remain consistent *overall*—that is, measured over all species in all clades at a given time.
4. If we further assume that rates of evolution are associated with body size and generation time (e.g., high rates being linked to small bodies and short generation times), we expect that a randomly chosen modern species will have experienced an increase in body size and generation time in the recent past, having probably originated from ancestors with smaller body size and shorter generation time (c.f., Berv and Field (2018)).

Each of these predictions already enjoys some degree of empirical support in the existing literature, as indicated above. However, further research is needed to test each systematically to the extent that these predictions could be judged to be successful or falsified.

In conclusion, our analysis suggests that a strong correlation between rates of molecular evolution and diversification would explain several empirical features of the natural world, unify two key areas of statistical modeling within a common framework, and point toward necessary developments in phylogenetic inference and molecular dating in which this link is made explicit, such as an extension of the CET model to permit direct inference of actual historical rates from molecular data.

## Data Availability

The R code for generating the figures is available from the Dryad Repository at  https://doi.org/10.5061/dryad.q573n5ts2

## Supplementary Material

Data available from the Dryad Digital Repository:  https://doi.org/10.5061/dryad.q573n5ts2

## Appendix 1

We will make extensive use of probability generating functions. A quick review of their important properties follows. A probability generating function, G(z) for the random variable X is defined as:

$$\begin{array}{ccc} G(z)=\underset{k}{\sum }P(X=k)z^{k} & & \end{array}$$
(A.1)

The probability generating function has several important properties that will be useful in the subsequent exposition. In particular:

1. Normalisation: $G(z=1)=\sum_{k}P(X=k)=1$ (in cases where P(X=k) represents a full probability distribution)
2. Extinction probability: G(z=0)=P(X=0)
3. Expectation: $E(X)=\sum_{k}kP(X=k)=\frac{\partial G}{\partial z}{|}_{z=1}$
4. Sum of random variables: If W=X+Y, then $G_{W}(z)=G_{X}(z)G_{Y}(z)$
5. Retrieval of probabilities: $P(X=k)=\frac{1}{k!}\frac{d^{k}G(z)}{dz^{k}}{|}_{z=0}$

In respect of point (5) above, the values of P(X=k) can be retrieved efficiently by Fourier inversion:

$$\begin{array}{lll} \begin{array}{lll} P(X=k) & =\frac{1}{k!}\frac{d^{k}G(z)}{dz^{k}}{|}_{z=0} & \\ & =\frac{1}{2}\int_{-\pi }^{\pi }G(\exp(i\theta ))\exp(-ik\theta )d\theta \end{array} & & \end{array}$$
(A.2)

Where the integral expression makes use of the Cauchy integral formula. This expression can be efficiently solved numerically using Fast Fourier Transform methods (Gleeson et al., 2014).

### Derivation of equation specifying evolution of the generating function

Define $G_{x}(t,z)=\sum_{n}P_{n}(t,x)z^{n}$ as the generating function for the number of species alive at time t from a process that starts at log-tempo x at time t=0. We indicate the x dependence by means of a subscript for reasons of notational clarity in later analysis.

Assume that we know the generating function for all x at some time t. How will the generating function change over a small increment of time Δt? Since the process is fundamentally homogeneous in time (i.e., there are no ‘special’ times’), we can construct this by considering a process that starts incrementally earlier than the known generating function. Within this small interval of time the process will change log-tempo incrementally according to an OU process, and furthermore may either speciate (producing two new independent processes with identical starting tempos) or go extinct. Given a current tempo x, the probability of speciation is $e^{x}\Delta t\lambda$, and that of extinction is $e^{x}\Delta t\mu$. Based on these possible events, the new generating function is given by a mixture of generating functions at time t:

$$\begin{array}{lll} \begin{array}{lll} & G_{x}(t+\Delta t,z)=G_{x}(t,z)+e^{x}\Delta t\int_{-\infty }^{\infty } & \\ & \left(\lambda G_{x^{\prime }}{\left(t,z\right)}^{2}+\mu -(\lambda +\mu )G_{x^{\prime }}(t,z)\right)p(x^{\prime }|x)dx^{\prime }. \end{array} & & \end{array}$$
(A.3)

Here $p(x^{\prime }|x)$ specifies the probability for the tempo to transition from x to $x^{\prime }$ over the time interval Δt. We take x to evolve via an OU process, with autocorrelation parameter θ and a stationary variance $s^{2}$, experiencing an effective time $e^{x}\Delta t$ within real time Δt. Given this specification we have:

$$\begin{array}{ccc} x^{\prime }|x∼N(x-\theta xe^{x}\Delta t,2s^{2}\theta e^{x}\Delta t) & & \end{array}$$
(A.4)

which yields: $E[x^{\prime }-x]=-\theta xe^{x}\Delta t$ and $E[{\left(x^{\prime }-x\right)}^{2}]=2\theta s^{2}e^{x}\Delta_{t}$ up to first order terms in Δt.

Taking a 2nd-order Taylor expansion of $G_{x^{\prime }}(t,z))$ around x and retaining first-order terms in Δt gives:

$$\begin{array}{lll} \begin{array}{lll} G_{x^{\prime }}-G_{x} & ≃e^{x}\Delta t\left(\left(\lambda G_{x}-\mu )(G_{x}-1\right)\right) & \\ & +E[x^{\prime }-x]\frac{\partial G_{x}}{\partial x}+\frac{1}{2}E[{\left(x^{\prime }-x\right)}^{2}]\frac{\partial^{2}G_{x}}{\partial x^{2}}. \end{array} & & \end{array}$$
(A.5)

Where we have dropped the explicit dependence of $G_{x}$ on arguments t and z for concision. Substituting the above expressions for $E[x^{\prime }-x]$ and $E[{\left(x^{\prime }-x\right)}^{2}]$ and taking the limit as Δt→0 gives the fundamental PDE of diversity evolution as given in equation 2.

$$\begin{array}{ccc} \frac{\partial G_{x}}{\partial t}=e^{x}\left((\lambda G_{x}-\mu )(G_{x}-1)-\theta x\frac{\partial G_{x}}{\partial x}+\theta s^{2}\frac{\partial^{2}G_{x}}{\partial x^{2}}\right) & & \end{array}$$
(A.6)

### Initial and boundary conditions

The most obvious question one can ask of this equation is: what is the probability that a process starting at log-tempo x will generate n species over time t? To answer this question we must solve equation 2 for different values of z, and use the Fourier inversion formula to retrieve the probability distribution $P_{n}(x,t)$. Solving equation 2 requires both initial and boundary conditions. For the question posed above the appropriate initial condition is given by G(t=0,x,z)=z∀ ⁡x, since a process that does not evolve for any time must have one species. Choosing appropriate boundary conditions is more difficult; since we must solve equation 2 numerically we take ’no flow’ boundary conditions ($\frac{\partial G}{\partial x}$ = 0) at some finite bounds $x_{\min}$ and $x_{\max}$ (we will usually use −10<x<10).

We can also ask how many species of log-tempo y will be produced at time t by a process that starts with log-tempo x at time t=0. Define the generating function of this distribution by $G_{x}^{y}(t,x,z)$. Some consideration will show that the time evolution of $G_{x}^{y}$ obeys the same PDE as that of $G_{x}$, but with a different initial condition. Since a process that starts with log-tempo x cannot instantaneously evolve to one of y≠x, we use the initial condition: $G_{x}^{y}(t=0,x,z)=\delta (x-y)z$, where δ(⋅) is the Dirac delta function.

### Evolution of the mean diversity

The mean of a distribution is straightforwardly recovered from its generating function via the relationship $E(n)=\sum_{n}nP_{n}=\frac{\partial G}{\partial z}{|}_{z=1}$. Applying this to the equation derived above for the evolution of the generating function gives the evolution of the mean diversity for a process that starts with log-tempo x. Defining $N_{x}(t)\equiv E(n|x,t)$ as the expected value of n at time t for a process starting with log-tempo x:

$$\begin{array}{lll} \frac{\partial N_{x}}{\partial t} & =\frac{\partial^{2}G_{x}}{\partial t\partial z}{|}_{z=1} & \\ & =e^{x}\left(2\lambda G_{x}{|}_{z=1}N_{x}-(\lambda +\mu )N_{x}-\theta x\frac{\partial N_{x}}{\partial x}+s^{2}\theta \frac{\partial^{2}N_{x}}{\partial x^{2}}\right) \end{array}$$
(A.7)

Since $G{|}_{z=1}=1\forall x,t$ by definition, we can simplify this to the expression given in equation 4:

$$\begin{array}{ccc} \frac{\partial N_{x}}{\partial t}=e^{x}\left(rN_{x}-\theta x\frac{\partial N_{x}}{\partial x}+s^{2}\theta \frac{\partial^{2}N_{x}}{\partial x^{2}}\right) & & \end{array}$$
(A.8)

where r=λ−μ.

By using initial conditions $N_{x}(t=0)=1\forall x$, solving this equation gives the mean number of species generated by a process starting at time t=0 and log-tempo x. As with the discussion of initial conditions above, we can also apply the same equation with different initial conditions to consider how many species with specific log-tempo y are generated by a process that starts at log-tempo x. Denoting the expected number of such species of this type as $N_{x}^{y}(t)$, in this case we use the initial condition $N_{x}^{y}(t=0)=\delta (x-y)$, analogously to the case of solving for the generating function. By definition, the expected number of species in total will be the sum over all final log-tempos: $N_{x}(t)=\int_{-\infty }^{\infty }N_{x}^{y}(t)dy$. Furthermore, we can ask what the expected number of species with log-tempo y is at time t if the starting log-tempo is unknown but specified by a probability distribution p(x). In this case we have:

$$\begin{array}{ccc} N^{y}=\int_{-\infty }^{\infty }N_{x}^{y}p(x)dx & & \end{array}$$
(A.9)

and the expected total number of species (considering all possible starting and current log-tempos) can be denoted simply as N(t) and is given by:

$$\begin{array}{ccc} N=\int_{-\infty }^{\infty }\int_{-\infty }^{\infty }N_{x}^{y}p(x)dxdy & & \end{array}$$
(A.10)

### Conditioning on survival

Equation 4 describes the evolution of the mean number of species through time, including all cases where the process goes extinct before the current time. If we want to ask how many species will be alive at time t, assuming that the process hasn’t gone extinct, we can do so straightforwardly by excluding the extinct cases:

$$\begin{array}{ccc} E(n_{t}|n_{t}>0)=\frac{E(n_{t})}{P(n_{t}>0)}=\frac{N_{x}(t)}{S_{x}(t)} & & \end{array}$$
(A.11)

where $S_{x}(t)=1-G_{x}(t,z=0)$ is the survival probability for a process starting at log-tempo x, determined from solving equation 2 for z=0. However, we may also want to know the expected number of species at some time t, conditioned on knowing that the process will survive to some future time T. In this case the conditioning is more complex. We make use of the identity:

$$\begin{array}{ccc} P(n_{t})=P(n_{t},n_{T}>0)+P(n_{t},n_{T}=0), & & \end{array}$$
(A.12)

which leads to:

$$\begin{array}{lll} \underset{n_{t}}{\sum }n_{t}P(n_{t}|n_{T}=0) & =\frac{\underset{n_{t}}{\sum }n_{t}P(n_{t})-\underset{n_{t}}{\sum }n_{t}P(n_{t},n_{T}=0)}{P(n_{T}>0)} & \\ \Rightarrow E(n_{t}|n_{T}>0) & =\frac{N_{x}(t)-C_{x}(t)}{S_{x}(T)}, \end{array}$$
(A.13)

where $C_{x}(t)$ is a correction term depending on x and t that we need to determine. Define a new generating function $H_{x}(t,z)=\sum_{n_{t}}P(n_{t},n_{T}=0)z^{n_{t}}$. Differentiating $H_{x}$ with respect to z and evaluating at z=1 gives the required correction term in the equation above. As with the generating function G, the evolution of H is governed by equation 2:

$$\begin{array}{ccc} \frac{\partial H_{x}}{\partial t}=e^{x}\left((\lambda H_{x}-\mu )(H_{x}-1)-\theta x\frac{\partial H_{x}}{\partial x}+\theta s^{2}\frac{\partial^{2}H_{x}}{\partial x^{2}}\right) & & \end{array}$$
(A.14)

Differentiating with respect to z gives:

$$\begin{array}{ccc} \frac{\partial C_{x}}{\partial t}=e^{x}\left((\lambda (2H_{x}{|}_{z=1}-1)-\mu )C_{x}-x\theta \frac{\partial C_{x}}{\partial x}+s^{2}\theta \frac{\partial^{2}C_{x}}{\partial x^{2}}\right). & & \end{array}$$
(A.15)

Unlike in the case for $G_{x}$, $H_{x}{|}_{z=1}$ varies as a function of x and t, and so solution of this equation for $C_{x}$ requires simultaneously solving this PDE and 2 with initial conditions: $H_{x}(t,z)=zG_{x}(T-t,z=0)$ and $C_{x}(t)=G_{x}(T-t,z=0)$.

### Lineages

Lineages are species in the past that have descendants in the present. Since molecular studies are based on extant species, any phylogeny reconstructed from these must consist of lineages. The evolution of lineages has thus been dubbed the ‘reconstructed process’ (Nee et al., 1994b), since these constitute the phylogeny that can, in principle, be reconstructed from molecular or morphological analysis of modern taxa.

We are interested in the number of species alive at time t which will have descendants at some later time T. Recall $N_{x}^{y}(t)$ is the expected number of species of log-tempo y at time t in a process that starts at log-tempo x. The expected number of these that will have descendants at time T is $S_{y}(T-t)$ (the survival probability over time T−t for a new process starting with log-tempo y). Thus the expected number of lineages of log-tempo y at time t is $S_{y}(T-t)N_{x}^{y}(t)$. Summing over values of y gives the total expected number of lineages, $M_{x}(t)$ at time t for a process starting with log-tempo x, viewed from the perspective of time T (we leave this dependence on the time of observation implicit in the notation, but note that lineages are only defined from the perspective of a specific point in time):

$$\begin{array}{ccc} M_{x}(t)=\int_{c\infty }^{\infty }S_{y}(T-t)N_{x}^{y}(t,x)dy & & \end{array}$$
(A.16)

This expectation includes the cases where the number of lineages is zero, i.e where there are no species at time T. If we wish to condition on the process surviving to the present we must remove these cases by dividing by $P(n_{T}>0)=S_{x}(T)$

$$\begin{array}{ccc} M_{x}(t)|[N(T,x)>0]=\frac{\int_{-\infty }^{\infty }S_{y}(T-t)N^{y}(t,x)dy}{S_{x}(T)} & & \end{array}$$
(A.17)

### Evolution of tempo distribution

Assuming that we start a process with log-tempo x, over time species generated by that process will diverge in tempos. How does this distribution of tempos evolve?

Consider starting a process with log-tempo x, and then selecting a species at random at some time t. The probability that this species has log-tempo y is given by:

$$\begin{array}{ccc} p(y|x,t)=\frac{N_{x}^{y}(t)}{\int_{-\infty }^{\infty }N_{x}^{y^{\prime }}(t)dy^{\prime }} & & \end{array}$$
(A.18)

If the starting log-tempo is unknown, but drawn from a distribution p(x), then we can marginalise the above equation with respect to x to find the later distribution p(y∣t):

$$\begin{array}{ccc} p(y|t)=\frac{\int_{-\infty }^{\infty }N_{x}^{y}(t,x)p(x)dx}{\int_{-\infty }^{\infty }\int_{-\infty }^{\infty }N_{x}^{y^{\prime }}(t)p(x)dxdy^{\prime }} & & \end{array}$$
(A.19)

Taking the derivative with respect to time gives:

$$\begin{array}{ccc} \frac{\partial p(y|t)}{\partial t}=p(y|t)\left(f(y)-\int_{-\infty }^{\infty }p(y^{\prime }|t)f(y^{\prime })dy^{\prime }\right) & & \end{array}$$
(A.20)

where $f(y)=\frac{1}{N^{y}}\frac{\partial N^{y}}{\partial t}$. That is, the distribution of log-tempos evolves according to a replicator equation, where the ‘fitness’ of a log-tempo y is given by the proportional increase in $N^{y}=\int_{-\infty }^{\infty }N_{x}^{y}p(x)dx$.

If we assume that at some point in time the distribution of log-tempos is given by p(x), we can consider the instantaneous evolution of $N^{y}$ from this time. Defining the current time to be t=0, we have the initial condition:

$$\begin{array}{ccc} N_{x}^{y}(t=0)=\delta (x-y) & & \end{array}$$
(A.21)

From equation 4, this implies that:

$$\begin{array}{ccc} \frac{\partial N_{x}^{y}}{\partial t}{|}_{t=0}=e^{x}\left(r\delta (x-y)-x\theta \frac{\partial \delta (x-y)}{\partial x}+s^{2}\theta \frac{\partial^{2}\delta (x-y)}{\partial x^{2}}\right) & & \end{array}$$
(A.22)

Applying standard rules for the operation of derivatives of the Dirac delta function, we can marginalise the above equation with respect to the initial distribution p(x) to give:

$$\begin{array}{lll} \frac{\partial N^{y}}{\partial t}{|}_{t=0} & =\int_{-\infty }^{\infty }\frac{\partial N_{x}^{y}}{\partial t}p(x)dx & \\ & =re^{y}p(y)+\theta \frac{\partial e^{y}yp(y)}{\partial y}+s^{2}\theta \frac{\partial^{2}e^{y}p(y)}{\partial y^{2}} \end{array}$$
(A.23)

Substituting this into equation A.32, and noting that again that $N^{y}=\int_{-\infty }^{\infty }N_{x}^{y}p(x)dx$, we get:

$$\begin{array}{ccc} \frac{\partial p(y)}{\partial t}=rp(y)\left(e^{y}-\langle e^{y}\rangle \right)+\theta \frac{\partial e^{y}yp(y)}{\partial y}+s^{2}\theta \frac{\partial^{2}e^{y}p(y)}{\partial y^{2}} & & \end{array}$$
(A.24)

Where $\langle e^{y}\rangle =-\int_{-\infty }^{\infty }e^{y^{\prime }}p(y^{\prime })dy^{\prime }$ is the mean value of $e^{y}$.

This then provides a replicator-mutation equation for the evolution of the tempo distribution, with the ‘fitness’ of log-tempo y being $re^{y}$. In particular, it specifies that the stable long term distribution of log-tempos is given by the solution to:

$$\begin{array}{ccc} rp(y)\left(e^{y}-\langle e^{y}\rangle \right)+\theta \frac{\partial e^{y}yp(y)}{\partial y}+s^{2}\theta \frac{\partial^{2}e^{y}p(y)}{\partial y^{2}}=0 & & \end{array}$$
(A.25)

Notably, we can see that if r=0, we recover the standard Fokker-Planck representation for the stationary OU process in the transformed distribution $e^{y}p(y)$:

$$\begin{array}{ccc} \frac{\partial e^{y}yp(y)}{\partial y}+s^{2}\frac{\partial^{2}e^{y}p(y)}{\partial y^{2}}=0 & & \end{array}$$
(A.26)

with the stationary solution p(y)= exp ⁡ (−s∕2)2πs2e−y exp ⁡  (−y22s2), implying a mean log-tempo of ⟨y⟩=−1.

From the equilibrium equation we can also find another useful relationship on the mean value. Multiplying equation A.37 by y and integrating gives:

$$\begin{array}{ccc} \int_{-\infty }^{\infty }y\left[rp(y)\left(e^{y}-\langle e^{y}\rangle \right)+\theta \frac{\partial e^{y}yp(y)}{\partial y}+s^{2}\theta \frac{\partial^{2}e^{y}p(y)}{\partial y^{2}}\right]dy=0 & & \end{array}$$
(A.27)

Integrating the partial differential terms by parts yields:

$$\begin{array}{ccc} (r-\theta )\langle ye^{y}\rangle -\langle y\rangle \langle e^{y}\rangle =0 & & \end{array}$$
(A.28)

from which we can see that if r=θ then ⟨y⟩=0, i.e. the mean log-tempo will converge to zero when the diversification parameter is equal to the mean-reversion parameter.

### Branch duration and expected molecular change

Considering a branch that begins with log-tempo x, what is the expected time until that branch terminates, either by speciation or extinction? For a branch to endure for time t+Δt it must first fail to terminate in time Δt, and then survive for a further time t with some new log-tempo $x^{\prime }$. Integrating over the possible values of $x^{\prime }$ we have:

$$\begin{array}{ccc} 1-F_{x}(t+\Delta t)=(1-e^{x}(\lambda +\mu )\Delta t)\int_{-\infty }^{\infty }(1-F_{x^{\prime }}(t))p(x^{\prime }|x)dx^{\prime }, & & \end{array}$$
(A.29)

where $F_{x}(t)$ is the cumulative probability that the branch originating with log-tempo x has terminated by time t.

Taking a second-order Taylor expansion of $F_{x^{\prime }}(t)$ around $x^{\prime }=x$ and retaining first order terms in Δt we have:

$$\begin{array}{ccc} \frac{\partial F_{x}}{\partial t}=e^{x}\left((\lambda +\mu )(1-F_{x})-\theta x\frac{\partial F_{x}}{\partial x}+\theta s^{2}\frac{\partial^{2}F_{x}}{\partial x^{2}}\right) & & \end{array}$$
(A.30)

The probability density for the branch to terminate at time t is given by differentiation of the cumulative distribution: $f_{x}(t)=\frac{\partial F_{x}}{\partial t}$. Applying this transformation to the equation above yields:

$$\begin{array}{ccc} \frac{\partial f_{x}}{\partial t}=e^{x}\left(-(\lambda +\mu )f_{x}-\theta x\frac{\partial f_{x}}{\partial x}+\theta s^{2}\frac{\partial^{2}f_{x}}{\partial x^{2}}\right) & & \end{array}$$
(A.31)

The probability density for a branch to terminate at time t thus follows the same form of differential equation as that for the mean number of species (equation 4), but with −(λ+μ) taking the place of r. Solving this equation requires the initial condition $F_{x}(t=0)=0\forall x$, which implies $f_{x}(t=0)=e^{x}(\lambda +\mu )$.

Assuming that molecular rates of change are covariant to tempo ($e^{x}$), for every increment of time Δt the expected amount of molecular change Δw (in arbitrary units that we label as myrs-equivalent; 1 myrs-equivalent being the expected molecular change in 1 myrs at a fixed tempo of τ=1) is $\Delta w=e^{x}\Delta t$. We can transform the above equation for $F_{x}(t)$ (which is given in terms of real time t) into one that applies over w via a change of variables, to give the cumulative probability $F_{x}(w)$ that a branch terminates before accumulating w units of molecular change.

$$\begin{array}{ccc} \frac{\partial F_{x}}{\partial w}=\left((\lambda +\mu )(1-F_{x})-\theta x\frac{\partial F_{x}}{\partial x}+\theta s^{2}\frac{\partial^{2}F_{x}}{\partial x^{2}}\right), & & \end{array}$$
(A.32)

and as above we obtain the probability density to terminate at w, $f_{x}(w)$, by differentiation: $f_{x}(w)=\frac{\partial F}{\partial w}$:

$$\begin{array}{ccc} \frac{\partial f_{x}}{\partial w}=\left(-(\lambda +\mu )f_{x}-\theta x\frac{\partial f_{x}}{\partial x}+\theta s^{2}\frac{\partial^{2}f_{x}}{\partial x^{2}}\right) & & \end{array}$$
(A.33)

Here we have the initial condition $F_{x}(w=0)=0\forall x$, which implies $f_{x}(w=0)=\lambda +\mu$. Consideration of this equation will show that the partial derivatives in x are initially zero and will remain zero for all values of w. Thus we can simplify the equation to:

$$\begin{array}{ccc} \frac{\partial f_{x}}{\partial w}=-(\lambda +\mu )f_{x}. & & \end{array}$$
(A.34)

The solution to this equation is straightforward and shows that w follows an exponential distribution with rate λ+μ:

$$\begin{array}{ccc} f_{x}(w)=(\lambda +\mu )\exp(-(\lambda +\mu )w) & & \end{array}$$
(A.35)

The notable feature of this density is that it does not depend on the starting log-tempo x, implying that the amount of molecular change in a branch is independent of tempo.

### Schematic for genetic encoding of tempo

Here we describe a simple model for how a genetic encoding of tempo can lead to the modified OU process we take as the basis for tempo evolution. Consider a binary string of n bases represented as ’1’ or ’0’, and define ρ as the proportion of bases that are ‘active’ – that is, encoded as ’1’. We assume that these bases mutate independently and neutrally, and with a rate that is covariant to the tempo $e^{x}$, such that the probability for each base to mutate in a small interval of time Δt is $qe^{x}\Delta t$.

If the number of active bases at time t is given by $n\rho_{t}$, then in the interval of time Δt the number of bases that mutate from ’1’ to ’0’ is binomially distributed as $B(qe^{x}\Delta t,n\rho_{t})$, and similarly the number mutating from ’0’ to ’1’ is binomially distributed as $B(qe^{x}\Delta t,n(1-\rho_{t}))$. If we take n to be large and Δt to be small these binomial distributions can be approximated by normal distributions, such that the number of mutations from ’1’ to ’0’ is normally distributed with mean $qe^{x}\Delta tn\rho_{t}$ and variance $qe^{x}\Delta t(1-qe^{x}\Delta t)n\rho_{t}$, and the number of mutations from ’0’ to ’1’ is normally distributed with mean $qe^{x}\Delta tn(1-\rho_{t})$ and variance $qe^{x}\Delta t(1-qe^{x}\Delta t)n(1-\rho_{t})$.

The change in the number of active bases is given by the number mutating from ’0’ to ’1’, minus the number mutating from ’1’ to ’0’. Given the results above, this change is also normally distributed. Taking the limit as Δt becomes infinitesimal (denoted dt) and retaining only terms first order in dt we have:

$$\begin{array}{ccc} d\rho ∼N(-e^{x}q(2\rho -1)dt,qe^{x}dt∕n) & & \end{array}$$
(A.36)

This is equivalent to the following form of stochastic differential equation:

$$\begin{array}{ccc} d\rho =-e^{x}q(2\rho -1)dt+\sqrt{e^{x}(q∕n)}dW & & \end{array}$$
(A.37)

where dW is an increment from a standard Wiener process, with mean zero and variance dt.

To specify a genetic encoding of the log-tempo, let us now define x=α(2ρ−1), where α is some arbitrary constant of proportionality, such that when half of bases are active this defines x=0. We can then rewrite the above equation as:

$$\begin{array}{ccc} dx=-2e^{x}qxdt+\sqrt{4\alpha^{2}e^{x}(q∕n)}dW & & \end{array}$$
(A.38)

Defining new variables θ=2q and $s^{2}=\alpha^{2}∕n$, we have:

$$\begin{array}{ccc} dx=-e^{x}\theta xdt+\sqrt{e^{x}2\theta s^{2}}dW & & \end{array}$$
(A.39)

which is precisely the modified OU process specified in equation 1. By taking α to be sufficiently large we can extend the boundaries of minimum and maximum values of x such that arbitrarily high or low values of x are possible within this model. We have assumed that n is large, and this assumption means that boundary effects around ρ=1 and ρ=0 can be safely ignored as these states are highly unlikely to occur under a random mutation process.

This then provides a schematic representation of how tempo could be genetically encoded in a manner that naturally leads to the modified OU process description that we employ in this paper. The purpose of this schematic is not to argue that this represents the actual genetic encoding of tempo in any specific details, but instead to illustrate how such an encoding would naturally give rise to the mean-reversion properties of the OU process, via the action of entropic forces. That is, the log-tempo tends to revert to the mean not due to any ecological mechanism, but simply because there are more possible encodings with x≃0 than those that encode more extreme values of x. One way in which tempo might influence rates in the way required by the CET model would be if it was encoded by a multilocus set of genes that influence body size, as body size appears to be associated with a syndrome of other features such as generation time and mutation rate (Martin, 2017). This encoding would satisfy the requirements of the CET model, although we would stress again that we have no formal commitment to it.
